# Versatile Oral Insulin Delivery Nanosystems: From Materials to Nanostructures

**DOI:** 10.3390/ijms23063362

**Published:** 2022-03-20

**Authors:** Mengjie Wang, Chunxin Wang, Shuaikai Ren, Junqian Pan, Yan Wang, Yue Shen, Zhanghua Zeng, Haixin Cui, Xiang Zhao

**Affiliations:** Institute of Environment and Sustainable Development in Agriculture, Chinese Academy of Agricultural Sciences, Beijing 100081, China; wangmengjie@caas.cn (M.W.); wangchunxin@caas.cn (C.W.); ren19801266562@163.com (S.R.); panjunqian@caas.cn (J.P.); wangyan03@caas.cn (Y.W.); zengzhanghua@caas.cn (Z.Z.); cuihaixin@caas.cn (H.C.)

**Keywords:** oral insulin, absorption barrier, nanodrug delivery system, bioavailability

## Abstract

Diabetes is a chronic metabolic disease characterized by lack of insulin in the body leading to failure of blood glucose regulation. Diabetes patients usually need frequent insulin injections to maintain normal blood glucose levels, which is a painful administration manner. Long-term drug injection brings great physical and psychological burden to diabetic patients. In order to improve the adaptability of patients to use insulin and reduce the pain caused by injection, the development of oral insulin formulations is currently a hot and difficult topic in the field of medicine and pharmacy. Thus, oral insulin delivery is a promising and convenient administration method to relieve the patients. However, insulin as a peptide drug is prone to be degraded by digestive enzymes. In addition, insulin has strong hydrophilicity and large molecular weight and extremely low oral bioavailability. To solve these problems in clinical practice, the oral insulin delivery nanosystems were designed and constructed by rational combination of various nanomaterials and nanotechnology. Such oral nanosystems have the advantages of strong adaptability, small size, convenient processing, long-lasting pharmaceutical activity, and drug controlled-release, so it can effectively improve the oral bioavailability and efficacy of insulin. This review summarizes the basic principles and recent progress in oral delivery nanosystems for insulin, including physiological absorption barrier of oral insulin and the development of materials to nanostructures for oral insulin delivery nanosystems.

## 1. Introduction

Diabetes is listed as one of the top ten diseases threatening human health in the world, and it is also one of the fastest-growing global health crises in the 21st century. In 2019, the morbidity of diabetic patients worldwide accounted for 9.63%, and more than 4.6 million people die of diabetes each year [1,2]. Diabetes is divided into autoimmune reaction-induced Type I (T1DM) and insulin resistance-induced Type II (T2DM) [3]. Patients with T1DM almost completely lose their insulin secretion function, which need exogenous insulin supplement. Patients with T2DM are resistant to insulin, that is, the blood glucose levels of the patients are not sensitive to insulin [4,5]. In the early stage of T2DM, patients can be treated by rational diet control, active exercise, and oral hypoglycemic drugs, such as metformin and α-glucosidase inhibitors. However, in the late stage of T2DM, the patients’ blood glucose levels can only be controlled by direct injection of insulin [6].

At present, the U.S. Food and Drug Administration (FDA) has approved more than 100 types of insulin products for the clinical treatment of diabetes. However, because all these products are injections, difficulties in operation caused by injections will bring a great psychological burden to the diabetics. More serious is that injections cause many physiological hazards to patients, such as hypoglycemic reactions, lipoatrophy, fat hypertrophy at the injection site, local allergic reactions, erythema, itching, abscesses, and induration [7,8,9].

In order to improve the adaptability of the patients and reduce the pain caused by injection, the development of noninvasive insulin administration has become the research goal of many medical and pharmaceutical researchers. In recent years, a large number of scholars have developed noninvasive insulin administration such as perinasal [10,11,12], sublingual [13,14], transocular [15,16,17,18,19], pulmonary [20,21,22], and rectum [23,24] methods. However, it is still difficult to achieve the expected therapeutic effects due to the low bioavailability, the inability to simulate the concentration gradient of normal human insulin, the safety of additives, and low therapeutic activity [25,26].

Oral administration, as the most acceptable mode of administration, is also the safest and most convenient mode of insulin administration. Its prominent advantage is that it can avoid complications and hypoglycemia at the administration site [27,28]. However, the bioavailability of oral insulin administration is less than 2% due to its high molecular weight, strong hydrophilicity, poor stability, and low tolerance against hydrolysis by proteases [29,30,31,32]. Moreover, oral insulin can play its role only passing through the physiological absorption barrier of gastrointestinal tract, which has become a difficulty in the development of oral insulin [33,34]. Enzyme inhibitors, permeation enhancers, and pH regulators have been added to improve oral bioavailability of the formulation. Among them, the most representative example was ORMD-0801, developed by the Oramed pharmaceutical company, which included permeation enhancers, soybean trypsin inhibitor, and calcium chelating agent. At present, it is in clinical phase III and has a bioavailability of 5–8%, but the safety and efficacy of its additives are still uncertain [35].

**Figure 1:**
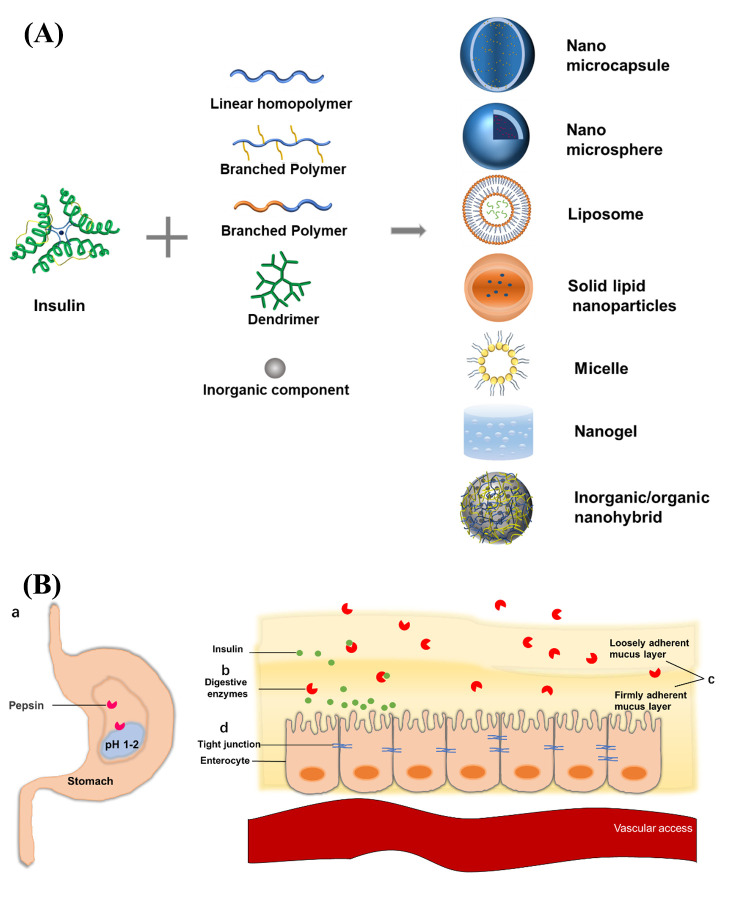
(**A**) Materials and nanostructures of oral insulin delivery systems. (**B**) The physiological absorption barrier of oral administration of insulin. (**a**) Destruction by gastric acid. (**b**) Degradation by digestive enzyme. (**c**) Retention by the mucus layer barrier. (**d**) Retardation by intestinal epithelial cell layer.

In the past 20 years, the combination of nanotechnology and pharmaceutics has brought possibility for the realization of oral administration of macromolecular drugs [36,37,38], and it is also a promising research direction of oral insulin [39,40]. In this review, we summarize the physiological absorption barriers of oral insulin and discuss the nano-drug delivery systems constructed from different materials (Figure 1). The bioavailability of oral administration of insulin is being enhanced by various types of strategies to construct different delivery nanosystems. Finally, the future research tendency for the further improvement of bioavailability is addressed. 

## 2. Physiological Absorption Barrier of Oral Insulin

Insulin injections not only cause a heavy psychological burden for diabetics, but also cause numerous physiological adverse effects. Oral insulin administration is the most desired manner. It has the characteristics of painless and strong adaptability and is in line with the physiological mechanism of insulin action. However, the challenge of oral insulin administration is the very low bioavailability. The reason is that oral insulin must overcome many obstacles caused by gastrointestinal environment before entering the circulation in order to achieve the expected therapeutic effects [41,42]. For the construction of oral insulin delivery nanosystems, delivery systems with different functions and structures need to be designed for these physiological barriers. Herein, we summarized the compositions of these barriers and the main ways to overcome them in Table 1.

### 2.1. Destruction by Gastric Acid 

Insulin remains in the stomach for about 2 h after oral administration. The pH values of gastric acid are about 1.2–2 [3,59,60,61]. The strong acidity in the stomach affects the ionization of amino acids and breaks the spatial structure of peptides and proteins [62]. Many delivery systems stably encapsulate insulin under acidic conditions to avoid the interaction with the acidic environment, while they can degrade or swell to release insulin in neutral conditions [43,44,45,46,47].

### 2.2. Degradation by Digestive Enzymes

There are many digestive enzymes that degrade proteins and peptides in the digestive tract (Figure 1b). As we know, the stomach is rich in pepsin, and the neutral to weakly alkaline intestine is rich in trypsin, chymotrypsin, elastase, and carboxypeptidase [63,64]. After naked insulin was incubated in simulated gastric and small intestinal fluids containing digestive enzymes at 37 °C for 2–3 h, less than 10% of the insulin retained activity or, even, all insulin lost its activity completely [65,66,67]. At present, insulin has been mainly encapsulated by carrier materials to protect it from degradation by digestive enzymes. The porous inorganic carrier materials were prepared to encapsulate insulin through the size difference between insulin and digestive enzyme, which could prevent the degradation of insulin by digestive enzyme. At the same time, the digestive enzymes could also be shielded by the hydrophobic interaction between the carrier materials and the digestive enzymes [48,49,50,51].

### 2.3. Retention by the Mucus Layer Barriers

The epithelial layer of the intestine is covered with a layer of electronegative mucus secreted by goblet cells (Figure 1c). The mucus is mainly composed of water, and also contains small amounts of glycoproteins, proteins, electrolytes, and lipids [68,69,70]. Electroneutral or electropositive substances are more likely to be adsorbed and retained in the mucus layer. However, studies have shown that strong electroneutral substances may be electrostatically embedded in the mucus layer, resulting in inferior permeability. Researchers have designed an electrically neutral delivery system with “mucus inert” or a polymer coating with charge reversal properties to improve the permeability of mucus in the system [52,53,54,55].

### 2.4. Retardation by Intestinal Epithelial Cell Layer

The intestinal epithelial cell layer is the physiological barrier for substances to enter the blood or lymphatic system from the gastrointestinal tract (Figure 1d). The intestinal epithelial cell layer consists of enterocyte, mucus-secreting cup cells, micro-folded cells (M cells), secretin-secreting enteroendocrine cells, and lysozyme-secreting Pan cells [71]. These epithelial cells are closely linked to form a barrier for protein peptide drugs to pass through and resist the invasion of harmful substances at the same time [72,73]. In addition to the paracellular pathway, the main challenges of the transcellular pathway of systemic circulation include barriers to apical endocytosis of cells, degradation of lysosomes upon entry into cells, and difficulties in release from the basolateral to the circulation [25,41,42,74,75]. Absorption enhancers are widely used in insulin delivery systems to increase the absorption of insulin in the gastrointestinal tract. Chitosan and other materials that can open tight connections have also been used to construct insulin delivery nanosystems. The use of specific recognition to increase active transport can also increase the oral bioavailability, such as folic acid pathways, bile acid pathways, and betaine transporters [31,46,56,57,58,59].

The above-mentioned barriers lead to the ineffectiveness of oral naked insulin for oral administration. For the therapeutic purposes, insulin must be protected from enzymatic and acidic damages to maintain its structure and activity before it enters the systemic circulation. These active insulins also must cross the mucus layer and can be absorbed by the intestinal epithelium. Therefore, the conditions that need to be met to realize the effect of oral administration of insulin include the following aspects: a. avoidance of degradation by digestive enzymes; b. resistance to destruction by gastric acid; c. mucosal permeability; d. epithelial cell permeability; and e. no toxicity to the body.

## 3. Oral Insulin Delivery Nanosystems

In order to overcome the above-mentioned oral absorption barriers of oral insulin, an oral drug delivery nanosystem was prepared by using nanotechnology and suitable carrier materials loaded with bioactive substances to improve the oral bioavailability of insulin. Delivery nanosystems are made by dissolving, dispersing, embedding, adsorbing, or coupling drugs into carriers to produce various nanoparticles, including nanoliposomes, nanosolid dispersions, polymer micelles, nanocapsules, nanospheres, microemulsions, and inorganic/organic hybrids [76,77,78,79]. They have various functions, such as small size, large specific surface area, strong adhesion and targeting, easy access to human cells to achieve high drug efficacy, elimination of biological barriers of drug action, maintenance of drug stability, various functional modifications, and sustained or controlled release of drugs. Compared with conventional drug formulations, oral nano-drug delivery systems have significant advantages in terms of improving bioavailability, extended drug half-life, and targeted delivery [80,81,82]. Therefore, oral delivery nanosystems are widely used in the development of oral formulations of insulin. The formulation combines insulin with a variety of materials with specific functions using nanotechnology to achieve the effect of improving the bioavailability of oral insulin. The drug delivery nanosystem realizes the functions such as resistance to gastric acid and digestive enzyme degradation, and penetration into the mucus layer and small intestinal epithelial cells. Herein, we summarize the development in oral delivery nanosystems for insulin from the dominating materials to nanostructures.

### 3.1. Materials for Oral Insulin Delivery Nanosystems

Carrier materials are used to load insulin to construct oral insulin delivery nanosystems. The desirable materials should have pH responsiveness, bioadhesion, biocompatibility, biodegradability, modifiability, and ease of processing, so as to maintain drug stability and improve bioavailability. A variety of polymers have been commonly used in the construction of oral delivery nanosystems (Table 2). They can be classified as natural polymers and synthetic polymers according to different sources. Common natural polymer carrier materials include proteins, chitosan, sodium alginate, hyaluronic acid, starch, and bile acid [83,84].

Synthetic polymers mainly include polylactic acid (PLA), poly (lactic-co-glycolic acid) (PLGA), and polycaprolactone (PCL). In addition, a number of inorganic materials or inorganic/organic hybrids have been used in the construction of insulin delivery nanosystems. Herein, we summarize representative carrier materials for oral insulin delivery nanosystems (Table 2).

#### 3.1.1. Polylactic Acid (PLA)

**Figure 2:**
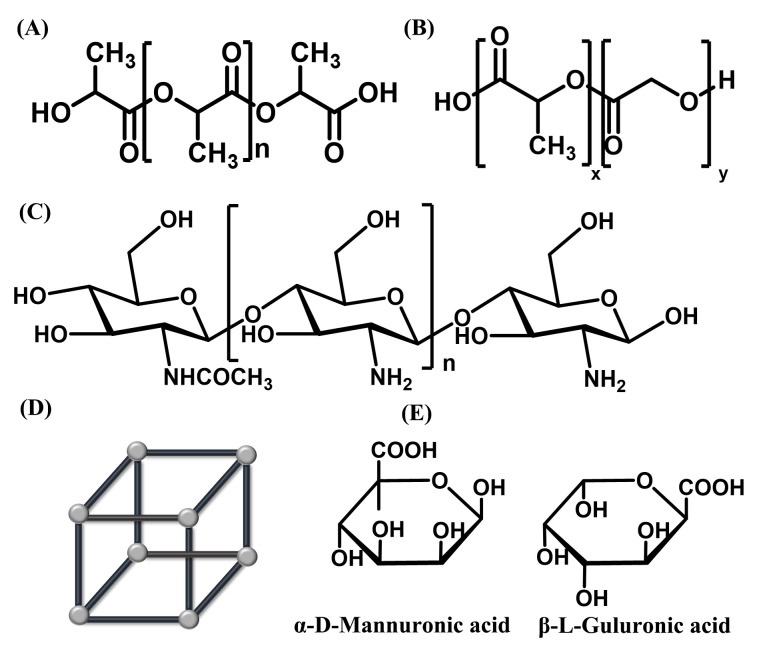
The chemical structures of the carrier materials for oral insulin delivery nanosystems. (**A**) PLA, (**B**) PLGA, (**C**) chitosan, (**D**) MOFs, and (**E**) alginate acid.

PLA is a kind of polyester derived from the polymerization of lactide as the main raw material (Figure 2A), which is biodegradable, biocompatible, and bioadhesive. PLA has been widely used in pharmaceutical formulations. PLA-*b*-Pluronic-*b*-PLA (PLA-F127-PLA) aggregates were synthesized to be used as nanocarriers for oral insulin [85]. The nanoparticle formulation maintained a hypoglycemic effect in diabetic rats for 18.5 h. The negatively charged hydroxyl and carboxyl groups of PLA increased adhesion to the intestinal wall and prolonged the residence time of nanoparticles, which was detrimental to the transport of the nanoparticles. PLA nanoparticles had an electrically neutral and hydrophilic shell after modification by an amphiphilic compound, lauryl phosphatidylcholine, which facilitated the diffusion of nanoparticles by shielding the negatively charged hydrophobic PLA cores and prevented prolonged adhesion to the mucus layer [104]. The amphiphilic polylactic acid insulin nanoparticles could reduce blood glucose by 40% within 4 h after oral administration compared with other PLA nanoparticles, which indicated that the modification of carrier materials could enhance the absorption of drugs after gastrointestinal uptake. Transportation of PLA nanoparticles could also be improved using a ligand-linked approach. PLA nanoparticles targeted the neonatal Fc receptor (Fc-RN) by coupling an immunoglobulin G crystallizable segment (IgG Fc fragment) to improve the bioavailability of insulin [86]. Adult Fc-RN expression levels were similar to those of the fetus and transport IgG antibodies through the small intestine and colon [87]. Enhancement could be observed on the basolateral side of the small intestine by fluorescent labeling, which indicated that these nanoparticles were transmitted and circulated through the intestinal epithelium. PLA has a high protective effect on insulin, because it is stable in gastric acid and is not prone to degrade. Insulin encapsulated by PLA needs to be dissolved with dichloromethane, which is the main reason why PLA cannot be widely used. At the same time, the degradation rate of PLA is too slow. PLA is not suitable for the preparation of quick acting insulin formulations, but more suitable for the preparation of long-acting and sustained release formulations.

#### 3.1.2. Poly (lactic-co-glycolic acid) (PLGA)

PLGA is a biodegradable functional polymeric compound formed by random polymerization of two monomers, including lactide acid and glycolide (Figure 2B). PLGA has good biocompatibility, nontoxicity, and good film-forming properties, and has been developed in oral delivery systems for macromolecular substances [105]. PLGA has a faster degradation rate than that of PLA, which is more suitable for the construction of oral insulin delivery systems. 

Insulin–phosphatidylcholine complexes were prepared by reverse micellar–solvent evaporation method in view of the poor water solubility of PLGA. The insulin complexes were loaded onto PLGA nanoparticles by a modified composite emulsion–solvent evaporation method, which improved encapsulation and permeability. First, a complex of sodium deoxycholate and insulin was formed by hydrophobic ion-pairing method and encapsulated into PLGA nanoparticles by emulsion–solvent diffusion method, which effectively improved the encapsulation rate (93.6%) and reduced the blood glucose level of diabetic rats to 43% of the original level [89]. Polymeric lipid hybrid nanoparticles consisting of a hydrophobic PLGA core, an amphiphilic phosphatidylcholine interlayer, and a hydrophilic PEG shell were constructed by spray freeze-drying. They were then filled into rigid gelatin capsules encapsulated with hydroxypropylmethylcellulose phthalate (HPMCP-55). The formulation exhibited good cellular internalization and the integrity of the drug encapsulation, which could maintain the integrity of drug encapsulation for up to three months [106]. The penetration of negatively charged PLGA nanoparticles through the mucus barrier was low. The surface modification of PLGA with positively charged compounds or targeted functional molecules can further improve the penetration rate [91,92]. PLGA nanoparticles modified with positively charged natural trimethyl chitosan (TMC) could facilitate the transportation of the nanoparticles [90]. Nanoparticles modified with TMC can penetrate into HT29 MTX cells and increase the diffusion rate by 28% compared with uncoated PLGA nanoparticles. However, the size of nanoparticles using PLGA as carrier is usually larger than 200 nm, which is not conducive to absorption. PLGA, as a material approved and certified by FDA, has good biocompatibility, no toxicity, no irritation, no immunogenicity, and sustained release, and still has a great potential in the field of drug research.

#### 3.1.3. Chitosan and Its Derivatives

Chitosan is a natural polymeric polysaccharide composed of deacetylated glucosamine and *N*-acetylglucosamine (Figure 2C). It has desirable biological properties, including biocompatibility, biodegradability, adhesion, and permeability [95,96,97,98]. The positive charge of chitosan interacts with the silicate group in mucin by hydrogen bond and electrostatic interaction, so as to enhance the adhesion of gastrointestinal tract [106]. The tight junction protein-4 (Claudin-4), an important protein, plays an important role in maintaining cell polarity and tight junction barrier function. Chitosan induces the redistribution of Claudin-4 from the cell membrane to the cytoplasm. Claudin-4 is then degraded by lysosomes, thereby weakening the tight junctions between cells and instantly increasing paracellular permeability. However, chitosan is insoluble under neutral and alkaline conditions. It is also difficult to protonate in the intestine to exert its cationic properties, which limits its absorption and utilization. Chitosan-derived compounds, such as quaternized chitosan, trimethyl chitosan (TMC), ethyl chitosan, carboxymethyl chitosan (DMEC), carboxymethyl chitosan (CMCS), acrylate-chitosan, and mercapto chitosan, were introduced to improve the aqueous solubility, adhesion, and permeability of nanoparticles at neutral and alkaline pH conditions. Their water solubility is higher than that of chitosan in a wide range of pH and concentration and does not affect their cationic properties. TMC is more likely to be aminated in neutral and alkaline environments, so as to improve its water solubility in alkaline environment and greatly increase the permeability of insulin. Because the protons of the primary amines of TMC are replaced by methyl groups, TMC can no longer form hydrogen bonds with the hydroxyl groups, so it is conducive to the absorption of hydrophilic compounds at a pH value similar to jejunum [107,108]. The most prominent advantages of chitosan and chitosan derivatives as materials for oral insulin delivery nanosystems are their strong adhesive properties and their natural positively charged properties. The obstacle of chitosan as insulin carrier is that it has certain toxicity to gastrointestinal tract. While chitosan opens up tight junctions, harmful substances can easily enter the blood through the paracellular pathway. On the other hand, the excessive positive charges tend to be retained in the mucus layer and cannot enter the circulation. In order to overcome the retention, some negatively charged polymers, such as alginate nanocompound and polyglutamic acid compounds, have been used to modify chitosan nanoparticles to improve the permeability across the mucus layer and the oral bioavailability.

#### 3.1.4. Metal Organic Frameworks (MOFs)

Metal organic frameworks (MOFs), also known as porous coordination polymers, are three-dimensional ordered porous materials consisting of inorganic clusters bridged by organic ligands. They have regular three-dimensional structure and stable porosity (Figure 2D), and their structures and chemical functions can be adjusted purposefully, which is widely used in drug delivery. Zhou et al. synthetized iron-based MOFs that could load insulin by physical adsorption, and the insulin-loaded MOFs could be coated with an amphiphilic polymer, poly (ethylene glycol-*b*-lactide), to keep it stable in the acidic environment of gastric juice [93]. Li et al. constructed a mesoporous zirconium-based MOF with a pore size of 3.2 nm and a loading capacity of up to 40%, which allowed insulin with a molecular size of 1.3 nm × 3.4 nm to enter the pores, while pepsin with a molecular size larger than 4 nm could not enter [95]. Therefore, the pepsin could not degrade the loaded insulin. Furthermore, this MOF was structurally stable under acidic conditions, preventing the release of the insulin in gastric juice, whereas in PBS, the structure of this MOF could disintegrate itself, thereby releasing the loaded insulin. However, the release rate of such drug delivery materials was too fast. About 80% of insulin was released in 40 min under physiological conditions, which may lead to side effects, such as hypoglycemia. At present, it is necessary to optimize and control the slow release of drugs. In addition, the degradation profile and metabolism pathway are still unknown, which might have potential negative effects on human heaths. Therefore, MOF materials for oral insulin delivery require further research.

#### 3.1.5. Other Materials

In addition, numerous other materials, such as natural polysaccharides and inorganic nanoparticles, are widely used in insulin oral delivery nanosystems. Anionic surface silica nanoparticles were designed to promote insulin absorption in the gastrointestinal tract. The negative electrical characteristics of the nanoparticle surface could induce the nanoparticles to relax the tight junctions among small intestinal epithelial cells by binding integrins and activating myosin light chain kinase (MLCK), increasing intestinal permeability and improving the uptake of nanoparticles by small intestinal epithelial cells. This effect is reversible and highly biocompatible and will not cause necrosis or inflammation of the intestinal tissues [39]. Ionic liquids are substances composed of ions with a melting point below 100 °C. They are liquid at or near room temperature, and are widely used in various fields, including pharmaceuticals. Banerjee et al. prepared a highly effective oral insulin formulation using choline and geranylate (CAGE) ionic liquids, which significantly reduced blood glucose to 45% of initial. The formulation exhibited excellent pharmacokinetic and pharmacodynamic results with good biocompatibility and storage stability for at least four months under refrigerated conditions [55]. Natural polysaccharides, such as sodium alginate and starch, are widely used in the oral delivery of insulin [95,101,102]. Sodium alginate is a byproduct of the extraction of iodine and mannitol from the brown algae kelp or Sargassum. Its molecular structure consists of β-D-mannuronic acid (β-D-mannuronic, M) and α-L-guluronic acid (α-L-guluronic, G) linked by (1→4) glycosidic bonds (Figure 2E), and is a hydrophilic, adhesive, biodegradable, biocompatible, and pH-sensitive natural polysaccharide. The guluronic acid can be cross-linked with divalent cations by exchanging sodium ions to form a gel matrix, in which hydrophilic drugs can be encapsulated. Insulin encapsulated in sodium alginate and chitosan by calcium chloride ionic gelation reduced blood glucose levels by more than 40% in diabetic rats at the doses of 50 IU/kg and 100 IU/kg, and the hypoglycemic effect lasted for more than 18 h. Insulin nanoparticles based on carboxymethylated short-chain amylose were constructed and coated with positively charged polysaccharides, which improved drug encapsulation rate and enhanced the permeation effect of nanoparticles in small intestinal epithelial cells, improved the absorption efficiency of insulin, and increased the bioavailability of insulin to 15.19% [104].

These above-mentioned materials provide a wide range of candidates for constructing nanostructures for oral insulin delivery. Based on these materials, nanotechnology is also an essential aspect of development of efficient oral insulin formulation. Thus, varieties of nanostructures have been intensively studied to improve the bioavailability of oral insulin drugs.

### 3.2. The Structures of Oral Insulin Delivery Nanosystems

The structure of oral insulin delivery nanosystems can be divided into liposomes, polymeric micelles, solid liposomal nanoparticles, organic nano-microspheres/microcapsules, nanogels, and inorganic/organic nanohybrids. The structures can be combined with varieties of functional components to improve their solubility, permeability, release properties, targeting, and protective effects. Herein, we summarized the structures of oral insulin delivery nanosystems.

#### 3.2.1. Liposomes

**Figure 3:**
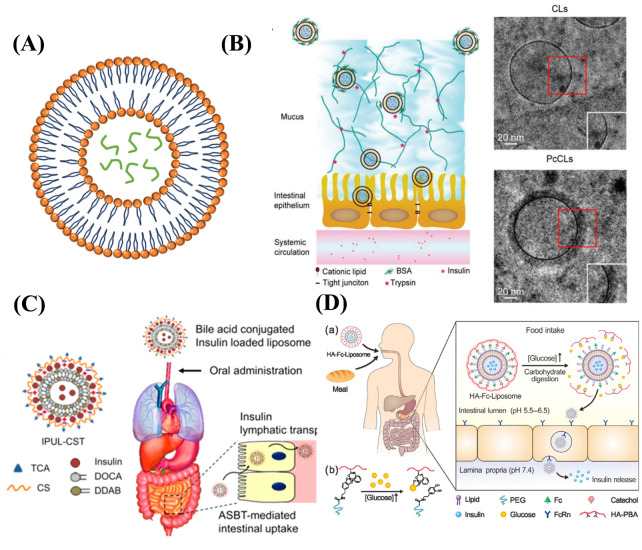
(**A**) The structure of liposomes. (**B**) TEM images of CLs and PcCLs, and schematic diagram for the process of the transport of the PcCLs through the mucus layers and epithelial barrier. (**C**) Schematic diagram of IPUL-CST and its intestinal uptake and lymphatic transport. (**D**) Schematic representation of the glucose-responsive oral insulin delivery liposomes for postprandial glycemic regulation.

Liposomes with the size ranging from 25 nm to 2.5 um are water-containing cored bilayer vesicles comprising phospholipid bilayer membranes (Figure 3A and Table 3). Liposomes have the advantages of low toxicity, high biocompatibility, good biodegradability, ease of scalability, reproducibility, and outstanding non-immunogenicity [109]. Once the liposomes enter the body, they will be regarded as an exogenous substance to stimulate the body’s immune mechanism. Then, they will be phagocytosed by the reticuloendothelial system and thus targeting enrichment in tissues, such as the liver, spleen, lung, and bone marrow, reducing toxicity to the heart and kidneys. However, liposomes aggregate in the gastric environment, and bile salts and trypsin lipase are prone to cause liposome degradation [110]. The physical stability of liposomes is poor, which is prone to produce laxatives and corruption. The commonly used lipid preparation methods include thin film dispersion, injection, ultrasonic dispersion, melting, and reverse evaporation [111]. Wang et al. prepared cationic liposomes (CLs) by thin film hydration using egg yolk lecithin (EPC), cholesterol, and the cationic lipid DOTAP as carrier materials [112]. Protein corona liposomes were prepared by adsorbing bovine serum albumin (BSA) on cationic liposomes in order to form neutral charge and hydrophilic surfaces to overcome mucus and epithelial barriers (Figure 3B). PcCLs could improve the oral bioavailability of insulin. In vitro and in vivo experimental studies have shown that the uptake and trans-epithelial permeability of PcCLs were 3.24 and 7.91 times higher than that of free insulin, respectively. Further studies on the behavior of PcCLs showed that when PcCLs crossed the mucus layer, the BSA corona could be shed from the PcCLs system, exposing CLs with positive electrical properties to promote epithelial uptake. Intra-jejunal injection of PcCLs had significant hypoglycemic effects in Type I diabetic rats, increasing oral bioavailability up to 11.9%. Kim et al. prepared an uncapped positive-charged liposomal nanoparticle (IPUL-CST) with a particle size of approximately 200 nm using a conventional thin film rehydration method [113]. The dimethyloctadecylammonium bromide (DDAB), deoxycholic acid (DOCA), and superparamagnetic iron oxide nanoparticles (SPION) with the diameter of 10 nm were used as materials (Figure 3C). Insulin was loaded by diffusion and electrostatic interaction into this uncapped special structure by dispersing superparamagnetic iron oxide nanoparticles in liposomes, allowing magnetic shear stress to squeeze the liposomal surface and tear it apart and forming open lipid bilayer pores. This allowed insulin to be encapsulated not only on the outside but also on the inside of the liposomes. The encapsulation rate of insulin in such nanoliposomes was significantly increased. The insulin-loaded liposomes were then encapsulated with a chondroitin sulfate-taurocholic acid coupling (CST). Complexation of the cationic liposomes with the anionic CST increased the active transport of IPUL-CST using the apical sodium-dependent bile acid transporter-mediated intestinal uptake and lymphatic transport pathways. It had shown that IPUL-CST absorbed via the distal ileum was delivered to the body’s circulation via the lymphatic pathway in vivo absorption pathway experiments. The apparent bioavailability of this insulin-loaded liposome after oral administration reached approximately 34%, and blood glucose consistently reduced at least 16 h after oral administration. This work was the first demonstration of an oral insulin delivery system directly triggered by increasing postprandial glucose concentrations in the intestine to provide an on-demand insulin release with ease of administration. Gu and his team reported a glucose-responsive nanoliposome with enhanced intestinal absorption function using phenylboronic acid conjugated hyaluronic acid (HA-PBA) shell coated with the (Fc Rn)-targeted liposome core (Figure 3D). This study demonstrates a responsive oral insulin delivery system, which is directly triggered by increasing postprandial glucose concentration in the intestine to provide on-demand insulin release and easy administration [40].

Liposomes as an oral insulin delivery nanosystem have outstanding biocompatibility, and some nanoliposome drugs have been approved for marketing, such as paclitaxel liposomes. Liposomes are widely used and have a high safety profile. However, the property of lipid materials results in low encapsulation efficiency. Due to the poor thermodynamic stability and short life of liposomes, nanoliposomes are more suitable for the preparation of quick-acting insulin preparations.

#### 3.2.2. Polymer Micelles

**Figure 4:**
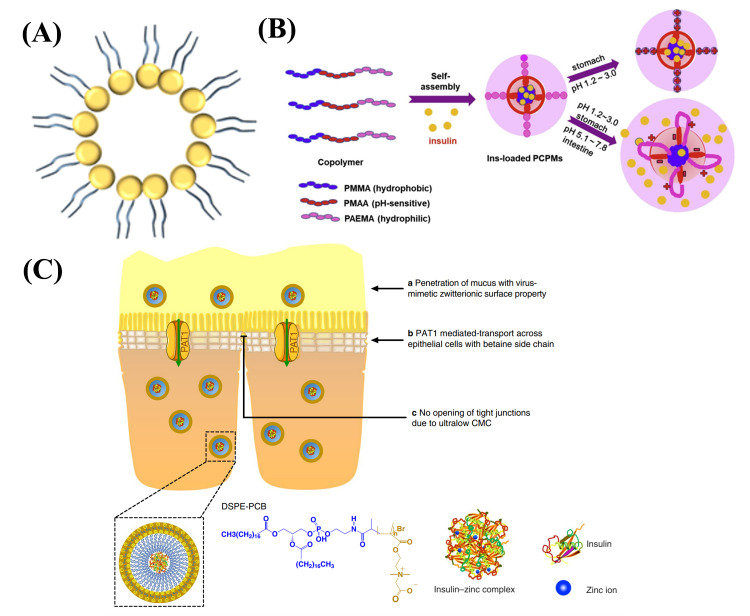
(**A**) Structure of micelles. (**B**) Schematic representation of Ins-loaded PCPMs and its pH-triggered release. (**C**) Schematic representation of DSPE-PCB micelles for oral delivery of insulin.

Polymer micelles are synthetic amphiphilic block copolymers, which can self-assemble in aqueous environments to form a thermodynamically stable colloidal system, which can spontaneously form polymeric micelles after dissolution in water because of their hydrophilic shell and hydrophobic core (Figure 4A and Table 4) [118]. The common materials for preparing polymer micelles are cohydrophilic blocks, such as polyoxyethylene, polyethylene glycol, polyvinylpyrrolidone, and hydrophobic materials, such as polylactic acid, methyl methacrylate, polystyrene, polypropylene, etc. [119]. Polymeric micelles protect insulin from sudden release and enzymatic degradation in gastric juice and then release in the intestinal environment, which can increase intestinal permeability and improve the efficiency of drug delivery. PH-sensitive polymer micelles can minimize the sudden release under acidic conditions in the stomach, which promote the adhesion of micelles and increase their residence time in the intestine.

Hu et al. designed pH-sensitive cationic polymer micelles with a core/shell structure that can be self-assembled in aqueous solution. The micelles were composed of methyl methacrylate (MMA, hydrophobic unit) and methacrylic acid (MAA, pH-sensitive and hydrophobic unit) as the core, with hydrophilic and pH-sensitive poly (2-aminoethyl methacrylate) (PAEMA) chain segments wrapped on the surface (Figure 4B) [47]. The PAEMA provided a spatial protective layer on the surface of the self-assembled micelles to enhance the stability of the micelles. The amine residues could be dramatically protonated in an acidic environment to form a positive-charged surface to provide an adhesive function, which improved drug permeability and bioavailability by opening the tight junctions of cells in the intestinal wall. Most polymeric micelles promote absorption by opening tight junctions among cells, but this approach also increases the invasion of harmful substances into the body. Han et al. used an amphoteric betaine polymer, DSPE-PCB (polymer (polycarboxybetaine, PCB) of 5000 Da molecular weight conjugated to 1,2-distearoyl-*sn*-glycero-3-phosphoethanolamine (DSPE)) to prepare DSPE-PCB micelles with a particle size of 25 ± 4 nm through mimicking the surface characteristics of chlamydial viruses to enable their rapid movement in the mucus layer (Figure 4C) [57]. These amphiphilic polymeric particles could enhance the bioavailability of insulin using the proton-assisted amino acid transporter 1 (PAT1) channel to facilitate the penetration of substances, such as betaine and betaine derivatives, into small intestinal epithelial cells. Transportation and in vitro experiments showed that the uptake of amphoteric micelles by PAT1 overexpressing cells (Caco-2) was increased significantly, while the uptake of amphoteric micelles was significantly inhibited in the presence of PAT1 substrate. In an in vitro fluorescence imaging study, DSPE-PCB micelles showed better retention and uptake in the small intestine compared to other micelles, such as polysorbate 80 micelles. Animal studies in diabetic rats showed that the bioavailability was as high as 42.6%. Monolayer micelles have smaller size and stronger permeability to small intestinal epithelial cells compared with liposomes. However, the drug loading rate of micelles was also relatively low. The high release rate and responsive release capability of micelles made them suitable for rapid postprandial glucose control.

#### 3.2.3. Solid Lipid Nanoparticles (SLNs)

**Figure 5:**
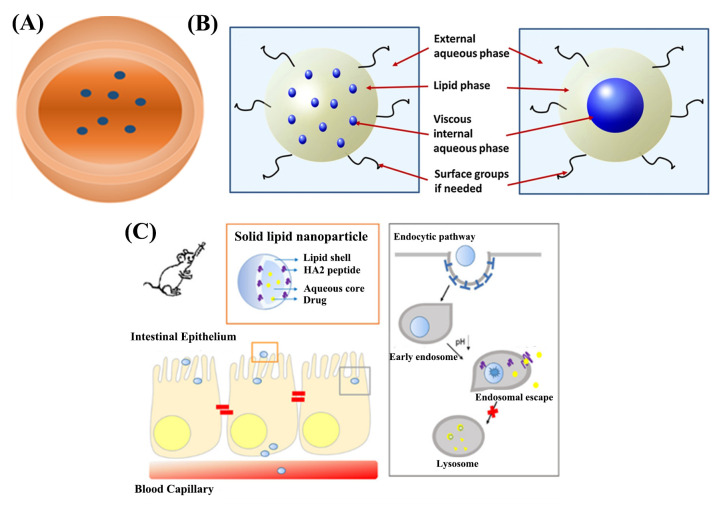
(**A**) Structure of SLNs. (**B**) Schematic representation of possible structures of VEN. (**C**) Schematic diagram of SLN and its behavior in intestinal epithelium.

SLNs are solid nanodrug delivery systems made of solid natural or synthetic lipids, such as lecithin, fatty acids, fatty alcohols, and other lipid-like materials, and the drugs are encapsulated or embedded in lipid-like nuclei (Figure 5A and Table 5). SLNs have low toxicity, no organic solvent, good biocompatibility, and high entrapment efficiency for hydrophobic substances. However, SLNs also have deficiencies, such as low encapsulation efficiency, short in vivo circulation time, and poor physical stability. The solubility of drugs in lipids and the limitations of preparation technology will lead to low drug content. SLNs can be prepared by high-speed homogenization, high-pressure emulsification, solvent emulsification, microemulsion, and ultrasonic dispersion [118,119,120,121,122,123,124].

Boushra et al. prepared SLN from soy lecithin. Viscosity-enhanced nanocarriers (VEN) were developed by adding a hydrophilic viscosity enhancer (VA) to the SLN core, which solved the problem of low encapsulation efficiency of hydrophilic active substances by SLN (Figure 5B) [125]. Oral insulin VEN showed good hypoglycemic effect in fasted rats with a relative bioavailability of 5.1%. Xu et al. prepared solid nanoliposomes with a shell containing endosomal escape factor hemagglutinin-2 peptide (HA2) by ultrasonication, which consisted of a soy lecithin solid lipid shell and an aqueous nucleus containing insulin [30]. The shell containing HA2 could effectively avoid lysosomal degradation of epithelial cells, the accumulation of insulin in the basolateral side of epithelial cells was much greater than that of free insulin, and the biological activity of insulin was maintained to a greater extent during intracellular transport (Figure 5C). SLN has better stability and simpler preparation method compared with liposomes. However, the encapsulation efficiency of hydrophilic drug insulin is still low. The development of oral insulin preparations still needs to be further studied.

#### 3.2.4. Organic Nanospheres/Nanocapsules

**Figure 6:**
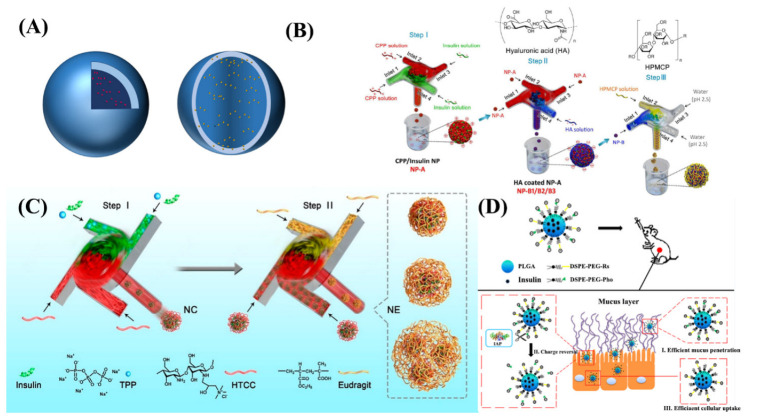
(**A**) Structures of organic nanospheres/nanocapsules. (**B**) Schematic representation of sequential FNC platform for preparation of the CPP/insulin nanoparticles. (**C**) The structure and the preparation process of NC-HTCC. (**D**) The structure of virus-like P-R8-Pho NPs and diagram of P-R8-Pho NPs to sequentially overcome mucus layer and intestinal epithelial cell layer.

Organic nanospheres and nanocapsules are spherical or encapsulated drug loaded particles with nanoscale diameters. They were prepared with natural or synthetic polymer materials as carriers by nanotechnology. Nanocapsules are composed of a polymer shell and a liquid (aqueous or oily) inner core, with the drug usually encapsulated in a polymeric membrane. Nanospheres, on the other hand, are homogeneous spherical solid drug delivery systems formed by mixing the drug in some way with a matrix of polymeric material (Figure 6A and Table 6). Compared to other nanocarriers, such as liposomes, micelles, emulsions, nanospheres, or nanocapsules, organic nanospheres can provide better storage and physiological stability to protect peptide molecules. They are prepared by emulsification–evaporation, nanoprecipitation, and self-assembly method [105,127,128,129,130,131].

He et al. used a transient nanoprecipitation technique (Figure 6B) with a hyaluronic acid-coated insulin/L-penetrating composite nanoparticle as the core and an enteric material hydroxypropylmethylcellulose phthalate (HPMCP) coating as the outer layer to prepare core–shell structured nanoparticles with controlled particle size, high encapsulation, and high drug loading rate, with a particle size of 45–115 nm and 11% bioavailability after oral administration [132]. Sun et al. used the FNC technique to electrostatically complex insulin with *a*-(2-hydroxy) propyl-3-trimethylammonium chloride modified chitosan (HTCC)/sodium tripolyphosphate (TPP) to form a nanocomplex (NC), followed by a secondary electrostatic complexation to further encapsulate the nanocomplex into the enteric material Eudragit L100-55, and prepared NC-HTCC of 87 nm particle size with better solubility and cell permeability under neutral conditions compared to normal chitosan (Figure 6C) [29]. The results indicated that the intestinal embedding method of peptide drugs endow drug formulations with better size controllability, batch reproducibility, and uniform surface coating properties, and significantly improved the oral bioavailability of insulin. Studies have shown that it would have great potential for clinical applications of oral protein therapeutics. Wu et al. constructed virus-like PLGA oral nanoparticles (P-R8-Pho NPs) with a particle size of 81.8 nm using a self-assembled nanoprecipitation method [53]. To further improve the mucus penetration and epithelial cell permeability of the nanoparticles, the surface of the nanoparticles was coated with oligoarginine R8 (a cell-penetrating peptide, rich in positively-charged arginine) and phosphatidylserine modifications. Brush border enzymes and intestinal alkaline phosphatase expressed by intestinal epithelial cells catalyzed the hydrolysis of phosphatidylserine, and then exposed positively charged R8, which makes the surface of nanoparticles positively charged and promotes the uptake of particles by small intestinal epithelial cells (Figure 6D). The nanoparticles could switch the surface charge for the different physiological environments of mucus and epithelial cell membranes, thus facilitating the permeation and absorption of the particles.

A large number of materials can be selected for the organic nanoparticle preparation process. Moreover, most of these materials for the preparation of nanoparticles have some reactive groups that facilitate further functional modifications, for example, functional molecules, such as linker ligands and phenylboronic acids, which allow the systems to achieve functions such as pH responsiveness, glucose responsiveness, and ligand–receptor-specific recognition. However, the biocompatibility of these materials still needs to be improved for the development of multifunctional insulin delivery nanosystems.

#### 3.2.5. Nanogels

**Figure 7:**
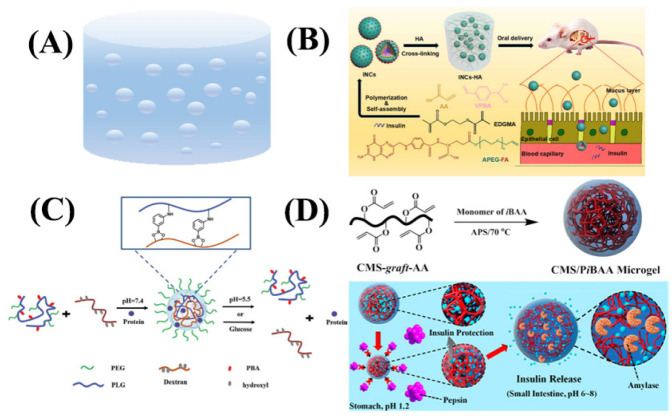
(**A**) Structure of nanogel. (**B**) Schematic representation of insulin-loaded glucose-responsive nanocarriers further encapsulated into hyaluronic acid (HA) hydrogel for oral delivery of insulin. (**C**) Schematic diagram of pH and glucose dual-responsive nanogels for protein delivery. (**D**) Synthetic process and its pH responsiveness of CMS/PiBAA hybrid microgel.

Nanogels are nanoparticles with a 3D network structure, produced by physical or/and chemical cross-linking of one or more hydrophilic monomers (Figure 7A and Table 7), which are rich in hydrophilic groups and can be swollen but not dissolved in water. The nanogels can be used as carriers to load hydrophilic insulin. After modification of the monomer polymer, the nanomaterials have the characteristics of sensitive release to pH value, temperature, and glucose [124,125,126].

Li et al. used the pH-sensitive monomer, ethylene glycol dimethacrylate (EGDMA), and the glucose-sensitive monomer, 4-vinylbenzeneboronic acid (VPBA), as materials to synthesize smart responsive nanogels of approximately 200 nm by microemulsion radical polymerization (Figure 7B) [134]. As the glucose concentration increased, hydrophilic phenylboronic acid–glucose complexes were formed, and the hydrogel size increased. Under the pH condition of the small intestine, the carboxyl group of acrylic acid lost its protons, which led to electrostatic repulsion between polymer chains. Nanogel produced a sparse gel structure and eventually released insulin from the nanoscale carrier. The system was further modified with polyethylene glycol–folic acid, which targeted the folate receptor on epithelial cells and promoted the penetration of the nanogel through receptor-mediated endocytosis. The hypoglycemic effect was verified in animal experiments. Si et al. developed a novel nanogel system with a particle size of approximately 44 nm based on poly (L-glutamic acid)-*g*-methoxypoly (ethylene glycol)/aminophenylboronic acid (PLG-*g*-mPEG/PBA) and dextran (Figure 7C) [133]. The nanogel was constructed through the reversible reaction of boron ester bonds between *cis*-diol on dextran and phenylboronic acid in PLG-*g*-mPEG/PBA, and insulin was loaded into the cross-linked lattice during the formation process. Since the boron ester bond broke at high glucose concentrations and weak acid environment, the prepared protein-loaded nanogels had good stability under normal physiological environments and could rapidly release insulin in weak acid and high glucose environments. It also had dual pH and glucose sensitivity. Effective endocytosis of the nanogels by cells could be observed by fluorescent imaging through confocal microscopy. Liu et al. used acrylic acid-grafted carboxymethyl starch (CMS-*g*-AA) and 2-isobutyl acrylate (iBAA) as monomers to prepare nanogels with a particle size of about 400 nm by aqueous dispersion copolymerization and loaded insulin into them by solubilization diffusion [41]. The system was pH-sensitive, with a mutation of ionization–deionization around pH 6.0 in the pKa value of iBAA, which conferred pH sensitivity to the material (Figure 7D). The accelerated breakdown of CMS-containing nanogels by amylase was confirmed by color development reactions and morphological changes, so that intestine-enriched alpha-amylase could degrade CMS to further accelerate insulin release in the intestine. This kind of nanomaterials could promote transmembrane transport of insulin into Caco-2 cells and enhance the oral pharmacological bioavailability of insulin.

The nanogels are highly hydrophilic and biocompatible, thus avoiding the elimination of immune system and maintaining long-term circulation. The main disadvantage of hydrogels is their poor storage stability, which makes it difficult to maintain drug persistence.

#### 3.2.6. Organic/Inorganic Nanohybrids

**Figure 8:**
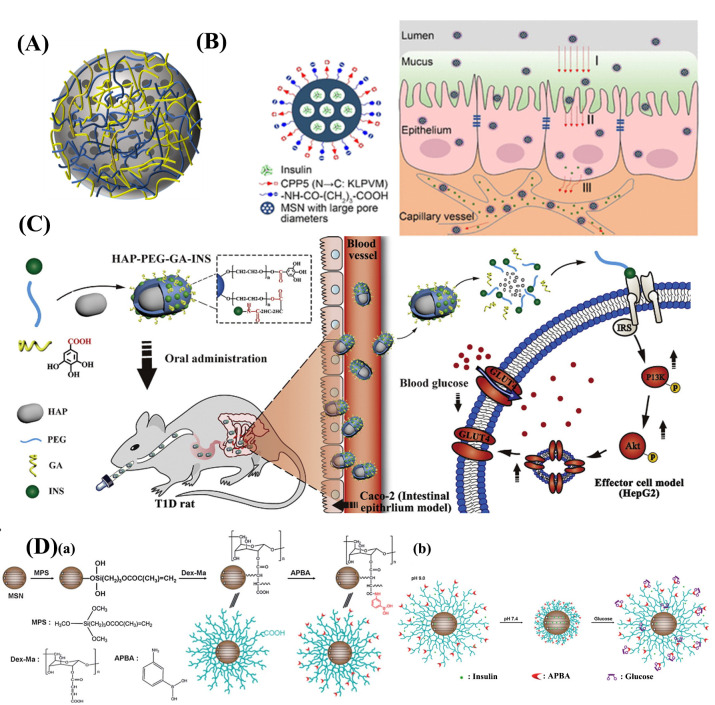
(**A**) Structure of inorganic/organic hybrid. (**B**) Structure of penetration behavior of virus-mimicking nanoparticles (MSN-NH2@COOH/CPP5). (**C**) Structure of HAP-PEG-GA-INS NPs and schematic diagram of insulin delivery to the effector cells by HAP-PEG-GA-INS NPs. (**D**) Structure and synthesis of the MSNs core–shell nanoparticles (**a**) and its pH- and glucose-sensitive behavior (**b**).

Organic/inorganic nanohybrids were composed of organic and inorganic materials by surface functionalization, one-pot synthesis, and wrapping (Figure 8A and Table 8) [130,131,132,133]. Inorganic materials as drug carriers are usually more stable and biologically inert compared with organic materials and provide better protection for drugs. However, inorganic materials are less functional. The wide use of organic materials offers a variety of options for the construction of nanohybrids. It can impart inorganic specific functionality and improve the dispersion and stability of nanomaterials.

Common materials include mesoporous silica, alumina, zirconium phosphate, and hydroxyapatite. Mesoporous silica nanoparticles are widely used because of their good biocompatibility, modifiable outer surface, and tunable pore size. Zhang et al. prepared insulin nanoparticles by introducing a membrane-penetrating peptide on the surface of mesoporous silica nanoparticles to mimic viruses [135]. Compared to nanoparticles with positively charged surfaces, such nanoparticles that mimic the surface structure of viruses could penetrate the mucus layer and reduce retention in the mucus layer (Figure 8B). These nanoparticles enhanced the efficiency of endocytosis through a cellular membrane cavity-like invagination mechanism, which further enhanced the ability to cross the small intestinal epithelial cells. Animal studies showed that this delivery system was able to reduce blood glucose levels by 50%, demonstrating excellent therapeutic efficacy. Zhang et al. prepared mesoporous silica nanoparticles of approximately 400 nm in size by coating polyethylene glycol on the surface of hydroxyapatite as a nucleus, and then coupling insulin and gallic acid with PEG (HAP-PEG-GA-INS NPs) (Figure 8C) [49]. Sun et al. coated the pH-sensitive material dextran-maleic acid and the glucose-sensitive material 3-aminophenylboronic acid on the surface of mesoporous silica nanoparticles (MSNs) for performance optimization (Figure 8D) [136]. The results of these nanocarriers in vivo on diabetic rats showed that they were more stable in hypoglycemic effect and reduced the probability of adverse reactions (such as hypoglycemia). 

It is difficult to prepare nanohybrid materials by combining the functions of organic and inorganic materials. How to combine organic and inorganic materials, how to maintain dispersion during preparation, and how to modify the inorganic component in a controlled manner with organic materials are still challenging. These are issues that are worthy of in-depth study. 

With the development of nanotechnology, a variety of insulin-loaded nanostructures have been constructed, which improves the bioavailability of insulin and lays a foundation for the development of oral insulin.

## 4. Summary and Outlook

Oral insulin administration is one of the most ideal methods of administration in terms of reducing pain and improving compliance for diabetic patients. However, the bioavailability of oral insulin remains low because of the physicochemical properties of insulin and the physiological barriers against absorption in the human gastrointestinal tract, making it difficult to achieve high efficacy. Functional factors, such as permeation enhancers, enzyme inhibitors, and pH regulators, are added to natural insulin formulation to improve oral bioavailability, but the efficacy and safety of additives are controversial issues. Based on the development of materials science, materials with different physical and chemical properties are studied. A variety of insulin-loaded nanostructures have been constructed to improve the bioavailability of oral insulin through nanotechnology, providing a basis for the development of oral insulin. By combining these materials with nanotechnology, oral insulin delivery nanosystems with various functions and delivery modes have been developed. These oral insulin delivery nanosystems are designed to improve bioavailability and effectiveness through pH responsiveness, glucose responsiveness, small size, charge variation, and facilitation of absorption and adhesion. Through various characterization experiments reported in literature, it was proved that these nanosystems have significant advantages in avoiding gastric acid, breaking through the retention of the juvenile layer barrier, passing through the intestinal epithelial cell layer, and responsive release. Significant improvements in oral bioavailability were also demonstrated in animal studies.

While the progress is seen, the deficiencies of the current oral insulin delivery nanosystem should also be considered. The safety of materials used to construct oral insulin delivery nan-systems needs to be further verified, and bioavailability is still not optimal. Although a large number of nonclinical data have been reported, the clinical progress of oral nano-insulin technology has not been satisfied due to the difficulty of delivering proteins orally. In addition, the preparation process of oral insulin is still complicated, which is not conducive to cost-effective commercial production. There is little data on the storage stability of these preparations.

In general, oral insulin is certainly an active research area because of the large number of diabetics and the disadvantages of insulin injection. The multifunctional delivery nanosystems can effectively improve the oral bioavailability of insulin and provide a promising strategy for oral insulin delivery. However, there is still a long way to go for the transformation of oral insulin delivery nanosystems from laboratory to clinic. In the future studies, more attention should be paid to material safety, precise control of drug dose, feasibility of preparation process, and storage stability. If the insulin delivery nanosystems can overcome these challenges, diabetics could be liberated from the pain of insulin injections.

## Figures and Tables

**Table 1:** The physiological barriers of oral insulin administration and the mechanisms.

Physiological Barriers	Constitution	Mechanisms to Overcome	References
Destruction by gastric acid	Gastric acid, pH 1.0–2.0	pH responsiveness	[43,44,45,46,47]
Degradation by digestive enzymes	Pepsin, trypsin, chymotrypsin, elastase, and carboxypeptidase	Shielding effect, hydrophobic effect	[48,49,50,51]
Retention by the mucus layer barriers	Water, glycoproteins, proteins, electrolytes and lipids	Charge-reversing, “Mucus-inert” electroneutral surface	[52,53,54,55]
Retardation by intestinal epithelial cell layer	Tight junction, apical endocytosis, degradation of lysosomes, and basolateral to the circulation	Permeation enhancer, increase the active transportation	[31,42,56,57,58]

**Table 2:** Representative materials for construction of oral insulin delivery nanosystems.

Materials	Carrier Components	Method	Active Components	EE%; LE%	Size (nm); PDI	Zeta-Potential (mV)	In Vitro Release Study (Condition, Time, Insulin Release)	Dose (IU kg^−1^)	In Vivo Studies	References
PLA	PLA, F127 [(PLA-F127-PLA) aggregates)]	Self-assembly	-	-; -	56; -	-	pH 7.4, 2 h, 55%	50	BGL, 5 h ^a^, 25% ^b^	[85]
	PLA, PEG	Nanoprecipitation	IgG Fc	-; 0.5	63; -	−5.6	pH 7.4, 2 h, 60%, 10 h, 100%	1.1	BGL 7 h, 55%	[86]
PLGA	PLGA (9.5 kDa) PLGA (100 kDa)	Reverse micelle-solvent evaporation method	SPC	80~90; -	200; -	−17~−12	pH 1.2, 2 h, 45%; pH 6.8, 6 h, 65%	20	rBA, 7.7%	[87]
		Double emulsion solvent evaporation	SPC, DSPE-PEG (2000)	92.36; 2.4	176; -	−31.1	-	40	rBA, 12.2%	[88]
	PLGA (50:50 ^c^, 20 kDa)	Double emulsion method	*N*-Trimethyl chitosan	47.0; 7.8	247; -	45.2	SGF, 6 h, 54.6%; SIF, 6 h, 72.5%	20	rPA, 11.8%	[89]
	PLGA (50:50, 8 kDa)	Double emulsion method	TDCS, Tat (YGRKKRRQRRR)	58.95; 1.38	157; 0.220	41.8	pH 1.2, 6 h, 20%; pH 7.4, 48 h, 15%	10	BGL, 12 h, 40%; BGL, 36 h, 80%	[90]
	PLGA polymer (50:50; 20 kDa)	Double emulsion method	Folic acid, Chitosan	41; 6.83	252; 0.237	5.99	pH 1.2, 6 h, 32.2%; pH 7.4, 6 h, 34.9%	70	rBA, 7.77%	[91]
MOFs	Fe-based mesoporous MOF	Physical absorption	SDS	51.6; 35.0	100; -	−18.3	pH 7.4, 14 h, 50%; pH 6.8, 14 h, 20%; pH 5.4, 14 h, 0	50	rPA, 7.8%	[92]
	Zr6-based MOF	Physical absorption	-	-; 40	-	-	pH 1.29, 1 h, 10%; pH 7.4, 1 h, 91%	-	-	[93]
Chitosan	Chitosan, γ-PGA	electrostatic interaction	-	75; 15	250; -	25	pH < 7, 100% pH > 7.0, disintegrating	30	rBA, 15%	[94]
	Chitosan (100 kDa, 90%)	self-assembly method	Hyaluronic acids (200 kDa), Biotin	71.72; -	277; 0.06	−27.90	250 U/mL trypsin, 2 h, 30%	50	rBA, 4.6%	[95]
	Chitosan (365 and 222 kDa, 86% ^d^), alginate	Electrostatic interaction and Chemical cross-linking	-	78.3; -	104; -	3.89	pH 1.2, 2 h, 25%; pH 6.8, 2~14 h, 60%~70%; pH 7.4, 14~24 h, 80~85%	100	rBA, 8.11%	[96]
	Chitosan (200–300 kDa, 85%), snail mucin	Self-gelation method	-	92.5; 21.4	504; 0.185	31.2	pH 1.2, 2 h, 10%; pH 7.4, 10 h, 87%	50	rBA, 10.6%	[97]
	Chitosan (150 kDa, 85.8%)	Self-assembly method	SDS, L-Phenylalanine	93.4; -	131; 0.227	30.71	pH 1.2, 2 h, 45%; pH 6.8, 4 h, 82%	50	rPA, 5.8%	[51]
	Chitosan (29.80 kDa, 80.2%)	Chemical cross-linking	Pentaerythritol tetrakis (3-mercaptopropionate)	79.63; 19.82	220; 0.091	2.3	pH 2, 12 h, 96%; pH 5.3, 24 h, 92%	50	3 h, 50%	[98]
	Carboxymethyl chitosan	Ionic cross-linking method	L-valine, PBA	67; 9.8	190; -	-	SGF, 24 h, 16.6%; SIF, 24 h, 50.7%; pH 7.4, 24 h, 55.4%; pH 7.4 (10 mM), 24 h, 68%; pH 7.4 (20 mM), 24 h, 92%	75	rPA, 7.55%	[99]
Others	Alginate, dextran sulfate	Emulsification/internal gelation, polyelectrolyte complexation	low molecular weight chitosan, bovine serum albumin	30.7; 6.2	300; -	28.9	pH 1.2, 2 h, 35%; pH 5.5, 2–4 h, 100%; pH 7.4, 2~8 h, 100%	-	-	[100]
	Proanthocyanidins, short-chain glucans	Recrystallization	-	70.2; 3.5	100~200; -	-	pH 1.2, 8 h, 60%; pH 6.8, 8 h, 75%	100	rPA, 6.98%	[101]
	HPMCP	Spontaneous emulsification solvent diffusion method		90.8; 8.13	200; <0.27	−15~0	pH 3.0, 4 h, 8.2%; pH 6.0, 4 h, 39.7%; pH 6.8, 4 h, 77.4%; pH 7.4, 4 h, 82.0%	25	rBA, 8.6%	[102]
	Waxy corn starch (approximately 99% amylopectin), Chitosan (140 kDa, 90%),	Self-assembly	-	89.6; 6.8	311; 0.227	−43.7	pH 7.4, 8 h, 50%	50	rBA, 15.19%	[103]
	Silica	-	SiO_2_		20~100; -			10	rBA, 23.4%	[39]

^a^: The time from medication to glucose level testing; ^b^: hypoglycemic effect; ^c^: degree of deacetylation; ^d^: the ratio of poly (lactic acid) and poly (glycolic acid); PDI: poly dispersion index; EE%: encapsulation efficiency; LE%: drug loading efficiency; rBA: relatively bioavailability, rPA: relatively pharmacological activity; BGL: blood glucose level; SPC: soybean phosphatidylcholine; PBA: phenylboronic acid; DSPE-PEG(2000): 1,2-distearoyl-sn-glycero-3-phosphoethanol-amine-*N*-methoxy (polyethyleneglycol)-2000; SDS: sodium dodecyl sulfate; TDCS: *N*-trimethyl-*N*-dodecyl chitosan; γ-PGA: poly (γ-glutamic acid); HPMCP: hydroxypropyl methylcellulose phthalate; -: not given in the literature.

**Table 3:** Examples of liposomes as a nano structure for oral insulin delivery nanosystems.

Materials	Method	Active Components	EE%; LE%	Size (nm); PDI	Zeta-Potential (mV)	In Vitro Release Study	Dose (IU kg^−1^)	In Vivo Studies	References
DOTAP, EPC	Thin-film hydration method	BSA	28.7; 1.5	195; -	−10.9	pH 6.8, 6 h, 45%	75	rBA, 11.9%	[112]
DDAB, DOCA	Thin-film hydration method	CST, SPION	75; 33	194; -	-	pH 1.2, 2 h, 10–14%; pH 7.4, 25 h, 47%	20	rBA, 34%	[113]
Mpeg2000-DSPE, HSPC	Extrusion, thin film hydration method	FA, PEG	70; -	180; <0.2	−12.9~−4.0	pH 1.2, 1 h, 25%; pH 6.8, 1 h, 48%	50	rBA, 19.08%	[114]
EPC, CH, SA	Thin film hydration Method, alternating electrostatic deposition	PAA, FA-PEG-PAH	>88; -	250; <0.27	25.4	pH 1.2, 2 h, 15%; pH 6.8, 1 h, 25%; pH 7.4, 25 h, 75%	50	rBA, 20%	[115]
PC, DSPE-PEG2000, CH	Microfluidic technique, nanoprecipitation	Chitosan, HPMCAS-MF, PEG	91; -	363; 0.315	23	pH 1.2, 2 h, 1%; pH 6.8, 8 h, 25%	-	-	[116]
EP, CH, DOTAP	Thin-film hydration technique	Chitosan	87.5; -	439; -	29.9	pH 1.2, 50 h, 18.9%; pH 7.4, 50 h, 73.3%	250	-	[117]
EPC, DOPE, CH	Lipid film hydration method	Glucose-sensitive hyaluronic acid shell; Fc Rn	20.7; 17.1	94; -	−28.1	pH 2.5, 12 h, <10%; pH 7.4, 12 h, <10%	10	-	[40]

DOATP: *N*-[1-(2, 3-Dioleoyloxy) propyl]-*N*,*N*,*N*-trimethylammonium methyl-sulfate; EPC: egg phosphotidylcholine; BSA: bovine serum albumin; RB: relative bioavailability, PAA: poly(acrylic acid); DDAB: dimethyl dioctadecyl ammonium bromide; DOCA: deoxycholic acid; CST: chondroitin sulfate-*g*-taurocholic acid; SPION: superparamagnetic iron oxide nanoparticles; DSPE: distearoylphosphatidylethanola-mine; mPEG: methoxypolyethelene glycol; HSPC: hydrogenated soya phosphatidylcholine; FA: folic acid; PEG: polyethylene glycol; CH: cholesterol; PAH: poly(allylamine hydrochloride), SA: stearylamine; HPMCAS-MF (M grade fine powders, abbreviated as MF): hydroxypropyl methylcellulose acetate succinate; PEG: poly(ethyleneglycol), DOPE: dioleoylphosphatidylethanolamine.

**Table 4:** Examples of micelles as nanostructures for oral insulin delivery nanosystems.

Materials	Method	Active Components	EE%; LE%	Size (nm); PDI	Zeta-Potential (mV)	In Vitro Release Study	Dose (IU kg^−1^)	In Vivo Studies	References
P(MMA-*co*-MAA)-*b*-PAEMA	Electron transfer, atom transfer radical Polymerization and self-assembled	MAA.MMA. AEMA	-; 9.1	neutral pH 200; -	15–25	pH 1.2, 10 h, 36%~40%; pH 7.4, 10 h, 50%~65%	-	-	[47,53]
PCB, DSPE-PCB		Zinc ion		25; -	−41	-	20	rBA, 41.2%	[104]
DODA-501, NIP AAm, AAC	Free radical polymerization		59; -	94~200; -		pH 1.55, 2 h, 45%; pH 7.4, 2 h, 60%	-	-	[120]

MMA: methyl methacrylate; MAA: methacrylicacid; AEMA: amino ethyl methacrylate; P(MMA-*co*-MAA)-*b*-PAEMA: Poly(methyl methacrylate-*co*-methacrylicacid)-*b*-poly(2-amino ethyl methacrylate); PCB: polycarboxybetaine; DSPE: 1,2-distearoyl-*sn*-glycero-3-phosphoethanolamine; DODA-501: dioctadecylamine-501; NIP Aam: *N*-isopropylacrylamide AAC: acrylic acid.

**Table 5:** Examples of SLNs as a nanostructure for oral insulin delivery nanosystems.

Materials	Method	Active Components	EE%; LE%	Size (nm); PDI	Zeta-Potential (mV)	In Vitro Release Study	Dose (IU kg^−1^)	In Vivo Studies	References
Soybean lecithin	double emulsion method	Peptide: GLFEAIEGFIENGWEGMIDGWYG	98.16; 7.52	161.6, 0.25	−16.1	pH 5.5, 12 h, 50%; pH 6.8, 12 h, 70%	50	rBA, 5.47%	[30]
Soy lecithin	Emulsification solvent-evaporation technique	propylene glycol	54.5; -	203.6, 0.175	−43.3	pH 2.5 (pepsin), 0.5 h, 40%	50	rBA, 5.1%	[125]
Glyceryl Trimyristate, Soya Lecithin	Double emulsification	L-penetratin	67.42; 1.82	745.3, 0.227	−23.7	pH 1.2, 6 h, 91%; pH 7.4, 6 h, 76%	10	rBA, 13.1%	[126]

**Table 6:** Examples of organic nanospheres/nanocapsules as nanostructure for oral insulin delivery nanosystems.

Materials	Method	Active Components	EE%; LE%	Size (nm); PDI	Zeta-Potential (mV)	In Vitro Release Study	Dose (IU kg^−1^)	In Vivo Studies	References
Poly(*N*-butylcyanoacrylate)	Self-polymerization	-	100; 20~60	120; -	−20–−10	pH 6.8, 2 h, 73.3%	50	rBA, 7.74%	[50]
Hyaluronic acid (190 kDa), HPMCP	FNC	Penetratin peptide (Ste-RQIKIWFQNRRMKWKK)	96.6; 66.7	103; 0.07	−19.7	pH 7.4, 12 h, 75%	80	rBA, 11%	[132]
PLGA	Self-assembly nanoprecipitation	DSPE-PEG2000-R8, DSPE-PEG2000-Pho	~35; -	81.8; 0.191	−2.39	pH 2.5, 0–2 h, 35%; pH 6.8, 2–8 h, 52%	50	rBA, 5.96%	[53]
Sodium tripolyphosphate, Chitosan (50 kDa, 95%)	FNC	*N*-(2-hydroxy)-propyl-3-trimethylammonium chloride modified chitosan	81.9; 35.6	106; 0.15	−24.6	pH 2.5, 0–2 h, 20%; pH 6.8, 2–8 h, 45%; pH 7.4, 8–24 h, 80%	80	rBA, 13.3%	[29]

HPMCP: hydroxypropylmethyl cellulose phthalate; FNC: flash nano-complexation.

**Table 7:** Examples of nanogel as a nanostructure for oral insulin delivery nanosystems.

Materials	Method	Active Components	EE%; LE%	Size (nm); PDI	Zeta-Potential (mV)	In Vitro Release Study	Dose (IU kg^−1^)	In Vivo Studies	References
(CMS-*g*-AA), iBAA	Aqueous dispersion copolymerization	Acrylic acid, carboxymethyl starch	-	pH 1.2, 480; pH 6.8, 700	-	pH 1.2, 4 h, 25%; pH 6.8, 4 h, 75%	60	rPA, 5.7%	[41]
PLG, dextran	Covalent cross-linking	PBA, PEG	44; -	43.7; -	−40	pH7.4, 72 h, 40.2% (Cg: 1 mg mL^−1^), 72.8% (Cg: 3 mg mL^−1^), 81.5%	-	-	[133]
EGDMA	-	VPBA, folic acid	68; -	166; -	-	pH 1.2, 0–2 h, 10%; pH 6.8, 2–8 h, 50%; pH 7.4, 0–24 h, 90% (Cg: 15 mM)	75	BLG, 5 h, 42.9%;	[134]

CMS-*g*-AA: acrylate-grafted-carboxymethyl starch; iBAA: 2-isobutyl-acrylic acid; PLG: poly (L-glutamic acid); Cg: the concentration of glucose; EGDMA: ethylene glycol dimethacrylate; VPBA: 4-vinylbenzeneboronic acid.

**Table 8:** Examples of inorganic/organic nanohybrid as nanostructure for oral insulin delivery nanosystems.

Materials	Method	Active Components	EE%; LE%	Size (nm); PDI	Zeta-Potential (mV)	In Vitro Release Study	Dose (IU kg^−1^)	In Vivo Studies	Reference
Mesoporous silica nanoparticles	Physical adsorption method	KLPVM peptide	80; 18	263.5; 0.175	−0.49	pH 6.8, 6 h, 40.52%	100	rBA, 2.84%	[135]
Hydroxyapatite, PEG	Homogeneous precipitation method, esterification reaction, amidation reaction	Gallic acid	45–60; -	150; -	30–40	-	50	-	[49]
Iron-based MOF, mPEG-b-PLLA, SDS	Oil/water emulsion	SDS, PEG	51.6; 35	~100; -	−18.33	pH 6.8, 12 h, 20%; pH 7.4, 12 h, 50%; pH 5.4, 12 h, 0%	50	rPA, 7.8%	[93]
Mesoporous silica nanoparticles	Aqueous polymerization and physical adsorption	APBA	77~89; 18~21	202.8; 0.078	−27.3	pH 1.2, 5 h, 15.2%; pH 7.4, 5 h, 18.8%; pH 7.4 (glucose 5 mM), 5 h, 80%	25	rBA, 3.1%	[136]
Porous silicon nanoparticles	Immersion method	Poly (pyridyl di-sulfide ethylene phosphate), Dodecyl sulfobetaine	~74; 10.3	241; 0.29	6.6	pH 1.2, 0~2 h, <1%; pH 6.8, 2~8 h, 35%	50	rBA, 4.36%	[137]

mPEG-*b*-PLLA: Poly (ethylene glycol)-block-poly(L-lactide); APBA: 3-amidophenylboronic acid.

## Data Availability

Not applicable.
